# A structural Model of Self-efficacy in Handball Referees

**DOI:** 10.3389/fpsyg.2017.00811

**Published:** 2017-05-18

**Authors:** Pierluigi Diotaiuti, Lavinia Falese, Stefania Mancone, Francesco Purromuto

**Affiliations:** Laboratory of Epidemiology, Physical Activity and Lifestyles, Department of Human Sciences, Society and Health, University of Cassino and Southern LazioCassino, Italy

**Keywords:** sport officiating, couple efficacy, teamwork, enjoyment, awareness

## Abstract

The study aimed to identify factors predicting self-efficacy in a sample of 248 Italian handball referees. The main hypothesis was that perception of teamwork efficacy would be a significant predictor of self-efficacy in handball referees. Participants completed an online questionnaire including *Referee Self-Efficacy Scale* (α = 0.85), *Self-Determination Scale* (α = 0.78), and an adaptation for Referees of the *Sport Commitment Model* (α = 0.80). Two hierarchical regression analyses have identified: (1) Enjoyment (β = 0.226), Couple Efficacy (β = 0.233), and Personal Awareness (β = 0.243), as predictors of Self-Efficacy; (2) Span of Co-Refereeing (β = 0.253), Perceived Quality of the Relationship (β = 0.239), and Mutual Agreement (β = 0.274), as predictors of Couple Self-Efficacy. A further SEM analysis confirmed the fit of a structural model of Self-efficacy considering the reciprocal influence of Couple Efficacy, Enjoyment and Awareness (χ^2^: 5.67; RMSEA: 0.000; SRMR: 0.019). The study underlines the importance of teamwork (or co-refereeing) as it relates to enjoyment and awareness in officiating and how it enhances the psychological well-being of handball referees. Future studies should investigate the relationship between factors influencing perceived teamwork efficacy and officiating performance outcome.

## Introduction

Referees and officials are a very important part of competitive sports not only because of their impact on players’ behaviors and game outcomes, but also because they ensure that competitions are conducted safely according to specific rules (Philippe et al., 2009; Warner et al., 2013). The tasks that referees perform are very complex given that they have to make quick decisions and solve conflicts, often accepting mistakes that might have been made as a result of their quick decision-making meanwhile keeping order and dealing with athletes, coaches, team managers, fans, and the media. (Rainey et al., 1990; Van Yperen, 1998; Anderson and Pierce, 2009; Guillén and Feltz, 2011). Cuskelly et al. (2008) define officiating as one of the most difficult and onerous tasks in organized sport. Concentration, speed, physical fitness, precision of reaction, anticipation, impartiality, and good cooperation with other officials are some of the skills required to perform their expected duties successfully (MacMahon et al., 2007; Pietraszewski et al., 2014). Aside from the factors involved in their regular tasks, environmental and external circumstances, like spectator reactions and atmosphere of the game, can also influence their performance and behavior. Psychological aspects such as self-confidence, concentration and emotional control play a determining role in referees’ behavior and decisions. Experienced referees declare that psychological skills influence up to 70% of their success (Weinberg and Richardson, 1990).

Due to the frequent criticism they receive for their decisions, referees are constantly under pressure with consequent anxiety, stress, and loss of confidence. These negative emotions that affect their mental health have been shown to be directly related to referee dropout, loss of attention, low performance, and low job satisfaction (Taylor et al., 1990; Goldsmith and Williams, 1992; Anshel and Weinberg, 1995; Rainey, 1995; Guillén and Bara, 2004; Guillén and Feltz, 2011; Nazarudin et al., 2014). Researchers have learned that a sense of community is very important in retaining referees and lowering dropout rates (Kellett and Warner, 2011). A study on volleyball referees found that enjoyment and commitment are highly important in preventing referees from developing intentions to quit (Van Yperen, 1998). According to Rainey (1999), age and burnout are two factors that can help predict the intention to quit refereeing. A study on referee burnout conducted in 2014 showed that less experienced referees are more prone to burnout than those with more experience (Al-Haliq et al., 2014). This burnout phenomenon is also very common in referees that experience emotional exhaustion.

Weinberg and Richardson (1990) assert that an effective refereeing measure could be based precisely on an individual’s ability to successfully handle different sources of stress. Alonso-Arbiol et al. (2005) found that the main sources of stress for referees are fear of failure or making a mistake, physical aggression and verbal abuse from players, coaches and/or the public as well as interpersonal conflict with coaches and players (Goldsmith and Williams, 1992; Rainey, 1995; Kaissidis-Rodafinos et al., 1997). Such verbal and physical abuse during and after a game has similarly been reported to be associated with negative influence on mental health (Rainey, 1999; Nazarudin et al., 2009).

The way referees perceive stress depends on experience and age (Kaissidis and Anshel, 1993; Rainey, 1995). Some studies suggest that younger officials reported to be more stressed than older officials regarding the possibility of making mistakes or calling fouls (Kaissidis and Anshel, 1993). Not only that, but when the source of stress is considered in relation to burnout, it has been noted that two specific sources of stress, specifically relationships with other referees and relationships with players could be considered especially important factors causing burnout (Garcés de Los Fayos et al., 1999).

More positive feelings of self-efficacy can help referees to boost their confidence in performing their tasks (Nazarudin et al., 2014). Referee self-efficacy is defined as the perception the referee has of his/her own capacity to perform tasks related to their job function (Guillén and Feltz, 2011). This seems to influence referee behavior, satisfaction, stress, and performance which, in turn, effects athlete rule violations and coach behavior. Studies on self-efficacy in sport show a negative association between the perception of low self-efficacy and task-related performance (Haney and Long, 1995; Stajkovic and Luthans, 1998). A lack of efficacy can also bring about a loss of attention, meaning more mistakes, slower reactions, higher stress and increased burnout (Guillén and Feltz, 2011).

General motivation theories of self-efficacy mainly focus on the concept of intrinsic motivation and show that the theme of “enjoyment” (Reeve et al., 1986) and the need of self-determination and competence (Deci, 1975) are often positively associated with self-efficacy. When enjoyment and self-efficacy were analyzed in a model predicting physical activity behavior, enjoyment resulted the strongest predictor (Lewis et al., 2016). This study also showed a positive influence on the enjoyment of physical activity and self-efficacy rates. Enjoyment has also proved to be an important component of the sport commitment scale. Staying in the sport is a consequence of commitment. Enjoyment in the sports activity performed is one of its sources along with personal investments, involvement alternatives and social constraints (Scanlan et al., 1993). Helsen et al. (1998) stated that in sport activities, enjoyment depends on the task and the type of activity (practice, leisure activity, competition, etc).

Research in sports psychology by Sweet et al. (2014) has combined self-efficacy and self-determination in one model integrating the two theories in a cross-sectional study about physical education. The model they built was demonstrated to be a good fit. A sense of self-awareness, a subscale of the self-determination scale found in psychological studies, can be also considered a factor associated with positive self-efficacy (Csikszentmihalyi, 2000), however, no studies have been conducted demonstrating this in referees specifically.

Communication and teamwork are also very important for referees. Effective communication with players, coaches, and other officials is essential to being a successful referee (Grunska, 1999; Guillén and Feltz, 2011). Communication between officials promotes coordination, an important aspect of teamwork. The referee’s social experience is directly correlated to the referee’s involvement and developing a sense of community, two factors that are key in determining the ability to overcome on-court stresses (Kellett and Shilbury, 2007; Kellett and Warner, 2011; Warner et al., 2013). These social connections and relationships with other referees can help them face abuse and ease the decision-making process (Kellett and Shilbury, 2007). Perceptions of the group’s efficacy, or what Bandura (1986) calls “collective efficacy”, is an important element in sustaining team members’ commitments which can also increase the feeling of personal efficacy, especially if the actions are viewed as successful (Gecas, 1989).

Competencies, duties, responsibilities as well as sources of stress vary according to sport and the level of competition (Goldsmith and Williams, 1992). The most studied sports with regards to the psychological aspects of referees are basketball (Alker et al., 1973; Fratzke, 1975; Rainey, 1999; Warner et al., 2013; Smid, 2015), football (or soccer) (Alonso-Arbiol et al., 2005; Bartha, 2006; Piffaretti, 2007; Wolfson and Neave, 2007; El Bakry, 2013; Pietraszewski et al., 2014), rugby (Cuskelly et al., 2008; Nazarudin et al., 2014), and volleyball (Van Yperen, 1998). To the best of our knowledge, few studies have been performed on handball, a team sport where two referees with equal authority are in charge of each game.

Macra-Oşorhean et al. (2012) investigated the psychological characteristics of handball referees and found the presence of high levels of rational and emotional intelligence in their sample. They also assert that stress resistance and impulse control are very important for handball referees, in particular. Higher perceived stress in handball referees was positively associated with the number of years officiating meaning that referees with more experience report higher grades of stress (Tsorbatzoudis et al., 2005). The authors explain this phenomenon by saying that more experienced referees are typically assigned to more demanding games. The importance of referees in-game coordination and communication is even higher in handball than in sports previously analyzed since the two referees are assigned together throughout an entire competition and often are matched up together for many years (Mascarenhas et al., 2005; Brand et al., 2006; Boyer et al., 2015). According to official handball rules, the two handball referees have equal authority, roles and physical demand and for this reason it is very important to work together to reach joint decision and mutual agreement in a final decision (The International Handball Federation [IHF], 2016).

Although the importance of individual psychological aspects for referees has been analyzed by previous scientific literature, only a few studies have focused on the psychological dimensions of referees as a team or a group. For this reason, we have decided to study handball referees as a unit or team since during official matches, two referees officiate with equal authority and teamwork, making communication and cooperation fundamental to their success.

The present study aimed to identify factors predicting self-efficacy in a sample of 248 Italian handball referees. The main hypothesis is that the perception of teamwork efficacy is a significant predictor of self-efficacy in handball referees. The study was conducted through the partnership with the Mediterranean Handball Confederation, which intervened in the officiating section of the Italian Handball Federation.

## Materials and Methods

Participants were recruited during sport season 2015/2016 through the Italian Handball Federation (FIGH), the organization in charge of referees’ training, sports rules, and championships organization. All the 350 active licensed referees of national and regional level were invited by email to fill in an online questionnaire. Compilations took place over 2 months. After a first contact email in which referees were invited to the compilation, 248 subjects (*K* = 248/350 therefore 71% of whole population) sent back their questionnaire, 222 males (89.5%) and 26 females (10.5%). The average age was 34 years (*SD* = 11.18) with a range that varies from 18 to 55 years. The average experience of refereeing was 13 years (*SD* = 9.76). Depending on the level, 86 (34.7%) were national top-level referees, 64 (25.8%) were national referees of second level, 98 (39.5%) were referees of regional level.

### Instruments

The protocol included the following tools:

(1) *Referee Self-Efficacy Scale* (REFS) (Myers et al., 2012), consisting of 13 items with 5 Likert answer modalities that assessed four factors of self-efficacy: game knowledge, decision making, pressure, and communication. Subjects are asked to indicate how confident they feel in performing referee job in a range where 1 correspond with a low and 5 with an high level of confidence. The instrument has demonstrated good reliability by presenting a Cronbach’s alpha coefficient equal to 0.85.
(2) *Self-Determination Scale* (SDT) (Sheldon and Deci, 1993) assess individual differences in the extent to which people tend to function in a self-determined way. It reflects two factors: (1) being aware of their feelings and their sense of self (Self-Contact), and (2) feeling a sense of choice with respect to their behavior (Choicefulness). The SDS is a short, 10-item scale, with two 5-item subscales. The first subscale is *awareness* of oneself, and the second is *perceived choice* in one’s actions. Items ask participants to estimate which of two statements feels more true of them, considering a 5-point scale where 1 corresponds to “Only A feels true,” and 5 to “Only B feels true,” For example, “What I do is often not what I’d choose to do” versus “I am free to do whatever I decide to do.” The scale has demonstrated good internal consistency by presenting a Cronbach’s alpha coefficient of 0.78.
(3) Van Yperen (1998) adaptation of the *Sport Commitment Model* (SCM) (Carpenter et al., 1993), which was originally designed to examine the reasoning for individuals to continue their participation within certain sports. This model breaks down commitment in sport to five key factors. These factors include level of enjoyment (positive affective response to the sport experience that reflects generalized feelings such as pleasure, liking, and fun), involvement alternative (attractiveness of the most preferred alternatives to continued participation in the current endeavor), personal investment (personal resources that are put into the activity which cannot be recovered if participation is discontinued), social constraints (expectations or norms which create feelings of obligation to remain in the activity), and involvement opportunities (valued opportunities that are present only through continued involvement); all of which exhibit an effect on the individuals commitment to a specific activity. Although SCM was primarily applied to the youth-sport domain, Van Yperen (1998) proposed a first adaptation of this tool in his study among Volleyball Referees, by substituting in the items the references to “sport practice” with the term “officiating.” A translated version of this SCM adapted for refereeing was used in our study with Handball Referees. *Enjoyment* was assessed by four items whose response categories ranged from 1 (not at all) to 5 (very much) and with Cronbach’s alpha 0.96; *Involvement alternatives* by three-item five-point scale ranging from 1 (never) to 5 (very often) and with Cronbach’s alpha 0.92; *Personal investments* by three-item five-point scale ranging from 1 (not at all) to 5 (very much) and with Cronbach’s alpha 0.81; *Social constraints* by three five-point scale items ranging from 1 (completely disagree) to 5 (completely agree) and with Cronbach’s alpha 0.71; Involvement opportunities by four five-point scale items ranging from 1 (not at all) to 5 (very much) and with Cronbach’s alpha 0.91.
(4) A *general questionnaire* that, in addition to socio-demographic information, collected data about:
  (1) main reason that accompanied the start of refereeing;
  (2) experience of co-officiating (duration and estimate of quality of the relationship over time measured through a 5-point scale ranging from 1 very bad to 5 excellent), (3) degree of couple agreement during officiating job (5-point scale ranging from 1 very bad to 5 excellent), (4) estimate of the efficacy of the officiating couple at the present moment (5-point scale ranging from 1 very bad to 5 excellent).

### Statistical Analyses

Preliminarily was considered the percentage distribution of motivation for arbitrage in the sample, the mean differences between groups considering Referee category and level of experience through Anova and *t*-test, Levene’ test for homogeneity of variance, Tukey’s HSD as *post hoc* test, with BCa 95% CI, and *p* < 0.05. Eta squared and Cohen’s d were the measures of effect size considered. Reliability and internal consistency of the scales were assessed using Cronbach’s Alpha coefficient. Bivariate and multivariate analysis were run using the software SPSS v.22. Considering the main objectives of the study, after Pearson’s correlations were performed two Hierarchical Regressions in order to identify the predictors of Self-Efficacy and Couple Efficacy among the sample. A Path analysis was lastly run using the software LISREL 8.80 aiming to assess the fit of a structural model that comprises and explain the influence of predictors on Self-Efficacy of referees. To test the adequacy of the model were considered the following eight indices: (1) the chi-square; (2) the relationship between the value of the chi-square and the degrees of freedom; (3) GFI (Goodness of Fit Index); (4) AGFI (Adjusted Goodness of Fit Index); (5) RMSEA (Root-Mean-Square Error of Approximation); (6) RMSR (Root Mean Square Residual); (7) CFI (Comparative Fit Index); (8) NNFI (Non-normed Fit Index).

## Results

### Motivation to Officiate

Considering the reasons given by participants to explain what most prompted them to undertake the handball referee activities, passion for this sport and have been in the past a handball player who did not wish to break away from the environment, were the reasons more frequent recorded (44.4 and 22.6%), followed curiosity (12.1%), personal challenge (11.3%), money (6.5%), and external involving (3.2%). Regret values (attractiveness of the most preferred alternatives) were significantly higher for the group motivated by economic reasons, significantly lower in the group motivated by passion and the one made up of former athletes of handball: *F*(5.246) = 3.909; *p* = 0.003; η^2^ = 0.14.

### Category and Level of Experience

Self-efficacy scores present significant differences in relation both to the Arbitrage category that to the level of experience. The national first-level referees have a level of self-efficacy significantly higher than that of the regional referees *F*(2,246) = 3.410; *p* = 0.04; η^2^ = 0.05. Referees belonging to the group with less experience (<4 years of officiating) have significantly lower levels of self-efficacy compared to all other groups *F*(3,246) = 7,507; *p* = 0.000; η^2^ = 0.16.

If we consider specifically the experience of co-officiating, significant differences emerge with different variable scores. The group with greater co-refereeing experience (>3 years) shows higher values of Enjoyment: *t*(245) = -3059; *p* = 0.003; *MD* = -0.358; *SE* = 0.117; BCa 95% CI = [-0.589, -0.126]; *d* = 0.50, higher values of Commitment: *t*(245) = -2637; *p* = 0.009; *MD* = -0.254; *SE* = 0.096; BCa 95% CI = [-0.444, -0.063]; *d* = 0.42, higher values of Couple Efficacy: *t*(245) = -2933; *p* = 0.004; *MD* = -0.228; *SE* = 0.078; BCa 95% CI = [-0.382, -0.074]; *d* = 0.55, and smaller values of Regret: *t*(245) = -2987; *p* = 0.003; *MD* = -0.429; *SE* = 0.144; BCa 95% CI = [-0.713, -0.145]; *d* = 0.50. Considering the mere chronological age, subjects older than 33 years recorded higher values of Awareness: *t*(245) = -2048; *p* = 0.043; *MD* = -0.194; *SE* = 0.095; BCa 95% CI = [-0.381, -0.006]; *d* = 0.34. Younger referees instead felt with greater intensity social constraints (i.e., expectations of friends, family, and colleagues) that pushed them to continue to officiate: *t*(245) = 2.316; *p* = 0.022; *MD* = 0.387; *SE* = 0.167; BCa 95% CI = [0.056, 0.718]; *d* = 0.38.

### Bivariate and Multivariate Analysis

**Table 1** reports Pearson’s correlation coefficients for the variables considered in the study.

**Table 1:** Pearson correlation matrix for all means of the scales (*n* = 248; ^∗∗^, correlation is significant at *P* < 0.005 2-tailed; ^∗^, correlation is significant at *P* < 0.001 2-tailed).

	SE	CE	CH	AW	EN	PI	SC	RE	CO	IQ	SP	QR	AG
SE	1												
CE	0.300**	1											
Sig.	0.001												
CH	0.239**	0.094	1										
Sig.	0.007	0.318											
AW	0.308**	0.129	0.481**	1									
Sig.	0.000	0.173	0.000										
EN	0.359**	0.164	0.223*	0.083	1								
Sig.	0.000	0.082	0.013	0.357									
PI	0.262**	0.180	0.127	0.076	0.402**	1							
Sig.	0.003	0.055	0.159	0.400	0.000								
SC	-0.105	0.033	-0.028	-0.192*	0.036	0.084	1						
Sig.	0.246	0.729	0.758	0.032	0.690	0.354							
RE	0.201*	0.117	0.129	0.156	-0.571**	0.295**	-0.041	1					
Sig.	0.025	0.213	0.155	0.085	0.000	0.001	0.655						
CO	0.189*	0.145	0.141	0.002	0.723**	0.364**	0.576**	-0.722**	1				
Sig.	0.036	0.124	0.119	0.984	0.000	0.000	0.000	0.000					
IQ	-0.093	-0.034	-0.042	-0.109	-0.166	-0.081	0.087	0.411**	-0.228	1			
Sig.	0.302	0.721	0.644	0.229	0.066	0.368	0.339	0.000	0.011				
SP	0.112	0.253**	0.052	0.039	0.246**	0.234**	0.044	0.120	0.186*	-0.147	1		
Sig.	0.218	0.007	0.569	0.665	0.006	0.009	0.628	0.184	0.040	0.104			
QR	0.291**	0.280**	0.084	0.196*	0.285**	0.386**	0.135	0.187*	0.286**	0.013	0.267**	1	
Sig.	0.001	0.003	0.358	0.030	0.001	0.000	0.136	0.039	0.001	0.890	0.003		
AG	-0.004	0.271**	0.020	0.159	0.031	-0.174	0.021	0.008	0.028	0.022	-0.052	0.098	1
Sig.	0.966	0.004	0.830	0.078	0.734	0.055	0.817	0.933	0.755	0.810	0.567	0.281	


*Legend: SE, Self-Efficacy; CE, Couple Efficacy; CH, Choice; AW, Awareness; EN, Enjoyment; PI, Personal Investment; SC, Social Constraints (expectations of family, friends, and colleagues); RE, Regret (Involvement Alternatives); CO, Commitment; IQ, Intention to Quit; SP, Span; QR, Quality of Relationship; AG, Agreement.*

Subsequently hierarchical regressions were performed in order to identify among the variables significant predictors of self-efficacy among handball referees.

The preliminary verifications of the regression assumptions excluded the presence of multivariate outliers. Mardia’s multivariate kurtosis index (273.56) was in fact below the critical value [p (p+2) = 288]; so the relationship between the variables can be considered substantially linear. Low co-linearity was indicated by the low VIF values (Variance Inflation Factor) < 2 and high tolerance values > 0.60. For verification of the assumptions on the residuals, the average between the standardized and raw residuals was equal to 0; the Durbin–Watson test had a value of 1.88 and was therefore indicative of the absence of autocorrelation.

**Table 2** reports the results of a hierarchical regression performed on referees’ self-efficacy used as the criterion variable. The choice and the order of introduction of the variables in the regression was determined by both theoretical reasons and the extent of the correlation coefficients with the self-efficacy. First has been inserted *Couple Efficacy*, that for the refereeing specificity of handball was considered a distinctive feature, then *Enjoyment with Officiating*, from the Sport Commitment Model and ultimately *Awareness* of the Self, from theoretical model of Self-determination. The three variables reported significant mid-level correlation coefficients with the self-efficacy (0.300^∗∗^; 0.308^∗∗^; 0.359^∗∗^) but no significant correlation between them. The total variance explained by the three predictors identified is 20%. The predictors explained, respectively, 9% (efficacy of the couple), 5% (enjoyment with officiating), 6% (awareness of self) of the total variance. As noted by the standardized beta, coefficients indicate that the weight of the variable *personal awareness* and the estimate of *couple efficacy* are substantially equivalent (0.243 and 0.233), while enjoyment with officiating is presenting a slightly lower influence (0.221).

**Table 2:** Hierarchical Regression Analysis considering *Self-Efficacy* as the variable criteria.

	*B*	SE *B*	β	*t*	*P*
***Step 1***				
Constant	2.811 (2.106, 3.517)	0.356		7.896	0.000
Couple Efficacy	0.291 (0.118, 0.465)	0.088	0.300	3.327	0.001
***Step 2***				
Constant	2.340 (1.558, 3.121)	0.394		5.931	0.000
Couple Efficacy	0.255 (0.084, 0.427)	0.087	0.263	2.946	0.004
Enjoyment	0.147 (0.032, 0.263)	0.058	0.226	2.533	0.013
***Step 3***				
Constant	1.680 (0.790, 2.569)	0.449		3.743	0.000
Couple Efficacy	0.226 (0.058, 0.394)	0.085	0.233	2.666	0.009
Enjoyment	0.144 (0.032, 0.256)	0.056	0.221	2.548	0.012
Awareness	0.190 (0.056, 0.324)	0.067	0.243	2.817	0.006


**R*^*2*^ = 0.08, adj. *R*^*2*^ = 0.07, *F*(1,226) = 9.676 for Step 1; Δ*R*^*2*^ = 0.15, adj. *R*^*2*^ = 0.13, *F*(2,226) = 9.811 for Step 2; Δ*R*^*2*^ = .20, adj. *R*^*2*^ = 0.18, *F*(3,226) = 9.028 for Step 3 (*p*s < 0.001).*

**Table 3** reports the results of a second hierarchical regression performed on Couple Efficacy used as a criterion variable. Order of introduction of the variables in the regression was determined considering the extent of the correlation coefficients with Couple Efficacy: first has been inserted *Span of co-officiating* (0.253^∗∗^), then *Quality of Relationship* (0.280^∗∗^), and ultimately *Agreement* (0.271^∗∗^). Among the three variables only Span and Quality of Relationship reported a correlation of 0.267^∗∗^ between them. The total variance explained by the three predictors identified is equal to 19%. The predictors explained, respectively, 6% (span of the officiating couple), 6% (perception of improvement in quality of the relationship), 7% (progressive overcoming of disagreements) of the total variance. As noted by the standardized beta, coefficients indicates that the weight of the variable Span of the officiating couple and the perception of improvement of Quality in their Relationship are substantially equivalent (0.227 and 0.220), while the progressive overcoming of the disagreement has a slightly stronger influence (0.274).

**Table 3:** Hierarchical Regression Analysis considering *Couple Efficacy* as the variable criteria.

	*B*	SE *B*	β	*t*	*P*
***Step 1***				
Constant	3.939 (3.831, 4.047)	0.054		72.337	0.000
Span	0.018 (0.005, 0.030)	0.006	0.253	2.766	0.007
***Step 2***				
Constant	3.500 (3.154, 3.846)	0.175		20.030	0.000
Span	0.014 (0.002, 0.027)	0.006	0.206	2.270	0.025
Quality of Relationship	0.115 (0.029, 0.202)	0.044	0.239	2.634	0.010
***Step 3***				
Constant	3.935 (3.506, 4.364)	0.216		18.174	0.000
Span	0.016 (0.004, 0.028)	0.006	0.227	2.597	0.011
Quality of Relationship	0.106 (0.022, 0.189)	0.042	0.220	2.513	0.013
Agreement	0.163 (0.061, 0.265)	0.051	0.274	3.181	0.002


**R*^*2*^ = 0.06, adj. *R*^*2*^ = 0.06, *F*(1,226) = 7.653 for Step 1; Δ*R*^*2*^ = 0.12, adj. *R*^*2*^ = 0.10, *F*(2,226) = 7.500 for Step 2; Δ*R*^*2*^ = 0.19, adj. *R*^*2*^ = 0.17, *F*(3,226) = 8.783 for Step 3 (*p*s < 0.001).*

Subsequently a SEM analysis was performed combining in one explanatory model the predictors of Self-efficacy and Couple Efficacy identified by previous regression analysis. The aim this path analysis was to test the fit of a structural model of self-efficacy considering the influencing effects of couple efficacy, enjoyment and awareness. Our hypothesis is indeed that in officiating handball, these act as main determinants on Referee’ self-efficacy. The model was then subjected to evaluation through the use of structural equation models run by the software Lisrel 8.80 (Joereskog and Soerbom, 1993). To test the adequacy of the model were considered the following eight indices: (1) the chi-square; (2) the relationship between the value of the chi-square and the degrees of freedom (χ^2^/*df*: values between 1 and 3 are considered acceptable); (3) GFI with values greater than 0.90 indicating a good fit of the model; (4) AGFI values greater than 0.85 indicating a good fit of the model; (5) RMSEA with values between 0 and 0.8; (6) RMSR more low values of 0.08 indicates a good fit of the model; (7) CFI values of at least 0.90 indicate an adequate fit of the model; (8) NNFI values of at least 0.90 indicate an adequate fit of the model (Byrne, 1998; Hu and Bentler, 1999; Schermelleh-Engel et al., 2003; Barbaranelli and Ingoglia, 2013).

The confirmatory verification of this model presented the following fit indices collected in **Table 4**:

**Table 4:** Goodness of Fit statistics.

	*X*^2^	*df*	*X*^2^/*df*	CFI	GFI	AGFI	NNFI	RMSEA	RMSEA 90% CI	SRMR
*N* = 248	5.67	15	0.38	1.00	0.99	0.97	1.14	0.000	0.000–0.000	0.019


**FIGURE 1:**
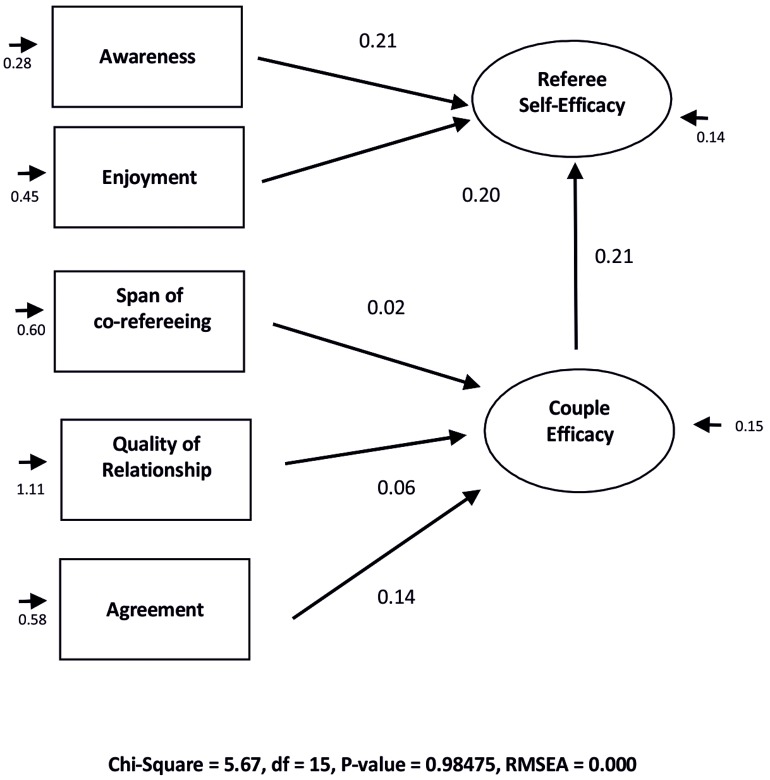
**Path analysis**.

The model shows good values of the fit. Overall, the model (See **Figure 1**) insists that to the component of Enjoyment with officiating, and that of Awareness of the Self, adds a further important component: the experience of co-refereeing, which in handball takes on special features that distinguish its officiating from those of the other sports. In this case, the span of common experience as officiating couple, the perception that over time the quality of the relationship and the mutual understanding of the couple were progressively improved, constitute the main elements that influence the judgment of referees about the efficacy of their own officiating couple.

## Discussion and Conclusion

The main findings of the current study confirmed the role of perceived of couple efficacy as a predictor for the perception of self-efficacy in handball referees. A preliminary explorative literature review led us to consider and assess variables related to Sport Commitment (Sport Enjoyment, Involvement Alternatives, Personal Investments, Social Constraints, and Opportunities Involvement) and Self-Determination (Self-Awareness and Perceived Autonomy) in the regression analysis. This analysis showed that sport enjoyment and self-awareness are also significant predictors of referee self-efficacy.

Self-efficacy is considered to be integral to successful experiences in sports (Feltz, 1988). Many studies have been done on self-efficacy in athletes (Ede et al., 2011) but very few have analyzed the psychological dimension of referees (Guillén, 2003). Some authors have identified the following as components for successful officiating: knowledge of the game, decision-making skills, psychological skills, strategic skills, communication/control of the game, and physical fitness (Guillén and Feltz, 2011). According to the scale developed by Myers et al. (2012) on the basis of the conceptual model by Guillén and Feltz (2011), beliefs of self-efficacy in referees of team sports include four sources: experience, significant others, physical and mental preparation, and partner qualifications. While most of these critical sources of self-efficacy have been investigated in previous studies, the role of what Guillen and Feltz call “significant others” has not been explored at length. “Significant others” are defined to include peers/partners, the main focus of this study and a considerably important component in self-efficacy.

We chose to analyze handball referees given that they always work with the same partner throughout competitions and often work together for many years. This characteristic led us to think that the perceived quality of the relationship between the two referees, considered a couple, could influence the perception of self-efficacy of each individual referee and consequently, his/her own performance and/or intention to quit.

Our analyses confirmed the hypotheses of a positive association between the perceptions of teamwork efficacy, or what we refer to as couple efficacy for handball referees, and the perception of each referee’s self-efficacy. In the hierarchical regression analysis where self-efficacy is considered the variable, we found a higher percentage of variance. In a further explorative analysis on couple efficacy predictors, we found that Span of Co-refereeing, Improvement of the Relationship, and Mutual Agreement are also significant predictors of Couple Self-Efficacy. In the structural model realized, the perception of couple efficacy, along with awareness and enjoyment, accounted for a high proportion of influence on the perception of self-efficacy.

To the best of our knowledge, there are no previous studies that investigate the relationship between teamwork and self-efficacy in handball referees but it is understood that cooperation, communication, support, respect, and trust are the basis of good teamwork for referees of sports in general (Richardson, 2007). Bad relationship with other referees is one of the sources of stress that could be considered an important factor for burnout (Garcés de Los Fayos et al., 1999). The importance of good communication and coordination between referees has been demonstrated in football by Boyer et al. (2015), explaining that when the coordination between central and assistant referees works well, the referee’s team performs better. For handball referees that have equal authority during a game, contrary to football, this concept becomes even more important for the decision-making process and good referee performance. The importance of teamwork was demonstrated in a non-sporting context showing that work-group cohesiveness and perceived task competence is associated with individual group member performance and organizational commitment (Wech et al., 1998). Ede et al. (2011) analyzed the importance of teamwork among dyads of athletes and coaches, or athletes and their teammates, and found that these relationships are also important for the development of an athlete’s self-efficacy.

For our sample of handball referees, the number of years refereeing together was very important in predicting the perception of a positive teamwork experience. Referees officiating as a team or couple for a higher number of years are more prone to experience commitment, enjoyment, and fewer regrets than their counterparts. Refereeing for a longer period of time also induces the perception of better relationships among the couple, more stability in the decision-making process and fewer disagreements.

The study also examined the relationships between referee self-efficacy and other variables such as refereeing level, years of refereeing experience, motivation, enjoyment and dimensions of self-determination. The findings show that age does not influence psychological skills, especially self-efficacy, results that are concurrent with the studies conducted by Anderson (2000). Previous studies found that more experienced referees reported less stress and higher self-efficacy (Guillén and Feltz, 2011). Similarly, Nazarudin et al. (2014) found no significant differences in psychological skills across age levels but significant differences across experience levels in rugby referees. These results are confirmed in our study where experience appears to be a predictor of self-efficacy, rather than age.

In previous studies, self-efficacy theories have been integrated with self-determination theories and have been considered as predictors of physical activity practices (Sweet et al., 2014). This integrated model proved to be a good fit for handball referees also. Within the SDTs, personal awareness is the factor that showed a higher predictability of self-efficacy in our study. Love for the game (of handball) was one of the most dominant reasons for becoming a referee. This result is in line with the findings of Burke et al. (2000) for basketball and Wolfson and Neave (2007) for football (or soccer). Referees that declared they started officiating for their love of the game or because they are former players reported a lower percentage of regret than those who indicated economical motivations as their reason for officiating. Regret is considered as the perception of losing opportunities and, according to Van Yperen (1998), a strong determinant of the intention to quit. Both enjoyment and involvement were found to deter the intention to quit officiating in volleyball referees which is why a positive affective environment and opportunities should be provided (Van Yperen, 1998). The perceived enjoyment in the activity performed, considered as an intrinsic motivation, has also been demonstrated as influential in self-efficacy ratings in a study done on physical activity (Lewis et al., 2016). Our study confirmed that this association exists also in handball referees.

Previous scientific literature provides interesting studies on self-efficacy in referees but we agreed with the review carried on by Lirgg et al. (2016) that more research was needed to assess the importance of the quality of the co-officiating experience. With this study, we have tried to fill a gap in the existing literature by exploring aspects such as teamwork, enjoyment, and self-determination, all of which have been demonstrated as being related to self-efficacy in other contexts outside of refereeing.

The main limitations of the present study include the gender composition of the sample (mainly male) and the fact that the measured variables are self-reported and don’t account a comparison with referees’ objective performance. Moreover, we only considered the perceptions of the referee as a single person independent from the perceptions of the pair as a unit therefore, the non-independence of data was not taken into account. Despite these limitations, the present research offers preliminary support and information to develop future studies and specific training programs for handball referees. In future research, it could be beneficial to deeply analyze predictors of self-efficacy considering the assessment of referee performance and mistakes during different game conditions.

In conclusion, we assert that some aspects of the referee experience, such as teamwork, enjoyment in officiating and dimensions of self-determination can provide a good explanation for perceived referee self-efficacy, despite them being little investigated by any of the previous literature. A better understanding of self-efficacy processes can help with the development of adequate intervention programs, which could improve the following outcomes: speed/accuracy of decisions, lower stress levels, less rule breaking, less dropout, greater satisfaction in the officiating experience and greater commitment (Lirgg et al., 2016). Enhancing positive affective responses found in enjoyment in officiating should be the focus of intervention programs further improving referee retention. For example, a mental preparation program targeting top-class referees was implemented by Piffaretti (2007) on the basis of an internationally approved scientific model for applied psychological intervention in sports (Weinberg and Gould, 1995) and proved to be an effective practical application of research findings.

Since we found that a positive teamwork experience plays a very important role in the psychological well-being of handball referees, having a proven impact on self-efficacy and also preventing dropout, future research should examine referees that officiate together as a team, analyzing their perceptions as a couple (in handball) and not only their perceptions on the couple (in handball). An investigation into the roles and commitment within the team, the influence of gender differences, mental models, leadership attitudes, and communication skills would be beneficial to explore in order to monitor the development process of the team of referees.

## Ethics Statement

This study was carried out in accordance with the recommendations of the AIP (Italian Psychology Association). All subjects gave written informed consent in accordance with the Declaration of Helsinki. The protocol was approved by Ethical Research Committee of the University of Cassino and Southern Lazio.

## Author Contributions

PD, LF, SM, and FP designed the study. PD, LF, and SM analyzed the data and discussed the results. PD and LF drafted the manuscript, and SM and FP revised the manuscript. All authors approved the final manuscript. Finally, the authors have agreed to be accountable for all aspects of the manuscript in ensuring that questions related to the accuracy or integrity of any part of it are appropriately investigated and resolved.

## Conflict of Interest Statement

The authors declare that the research was conducted in the absence of any commercial or financial relationships that could be construed as a potential conflict of interest.
